# Complete Genome Sequences of Microbacterium liquefaciens Phages Mercedes, Leafus, Nebulous, and Ixel

**DOI:** 10.1128/MRA.00068-21

**Published:** 2021-03-18

**Authors:** Victoria J. Frost, Kristi M. Westover

**Affiliations:** a Department of Biology, Winthrop University, Rock Hill, South Carolina, USA; DOE Joint Genome Institute

## Abstract

*Microbacterium* phages Mercedes, Leafus, Nebulous, and Ixel were isolated from soil in Rock Hill, SC. All are lytic phages with *Siphoviridae* morphotypes and similar genome sequence lengths that range from 40,200 bp to 42,000 bp. The four bacteriophages were isolated using the host Microbacterium liquefaciens.

## ANNOUNCEMENT

**FIG 1:**
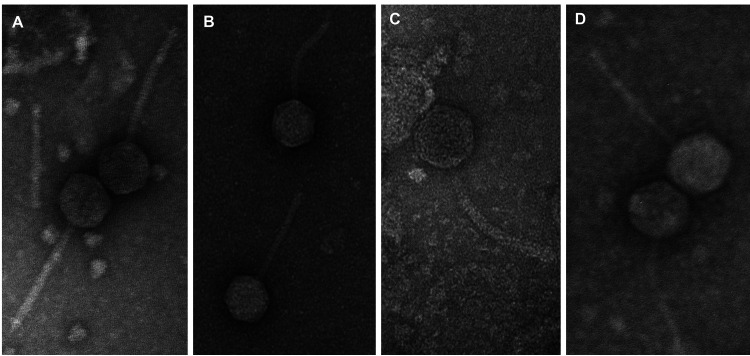
Transmission electron micrographs of *Microbacterium* phages Ixel (A), Leafus (B), Nebulous (C), and Mercedes (D). Phage lysates were negatively stained with 1% uranyl acetate.

An increase in the isolation and characterization of *Microbacterium* phages for potential therapeutic use, biotechnological applications, and host-pathogen evolutionary studies has recently occurred (1, 2). Here, Microbacterium liquefaciens LMG 16120 was used to isolate microbacteriophages in the soil at Winthrop University in Rock Hill, SC. This research is part of the Science Education Alliance-Phage Hunters Advancing Genomics and Evolutionary Science (SEA-PHAGES) program (3). Protocols were provided by the HHMI SEA-PHAGES discovery guide (https://seaphagesphagediscoveryguide.helpdocsonline.com/home). *Microbacterium* phages Mercedes, Leafus, and Nebulous were isolated directly from sandy soil under grass and flowers, while Ixel was isolated from deeper moist black soil (see Table 1 for global positioning system [GPS] location coordinates) and required an initial enrichment step with *M. liquefaciens*. All phage went through two rounds of purification and were amplified in the bacterial host grown on peptone-yeast-calcium agar (PYCa) medium at 30°C. Transmission electron microscopy revealed that all four phages had *Siphoviridae* morphologies with long flexible tails (Fig. 1). Phage DNA was extracted from high-titer lysates using the Wizard DNA cleanup kit (Promega) and sequenced at the University of Pittsburgh. Libraries were constructed using the NEBNext Ultra II FS DNA library prep kit and sequenced using the Illumina MiSeq v3 sequencing platform; 150-bp single-end reads yielded 1,094-fold (Mercedes), 1,489-fold (Leafus), 707-fold (Nebulous), and 1,263-fold (Ixel) coverage of each genome (Table 1). The reads were assembled using Newbler v2.9 and checked for accuracy, coverage, and genomic termini using Consed v29 as previously described (4, 5). The results (genome size, GC content, and predicted number of genes and termini) and accession numbers (GenBank and SRA) are listed in Table 1. Using an online tool (https://phagesdb.org/genecontent/) at the PhagesDB database (6), all phages were assigned by gene content similarity (GCS) into cluster EA (35% or greater GCS). Within the cluster, Leafus was assigned into subcluster EA1, Nebulous into subcluster EA5, and Ixel into subcluster EA11 (1, 7).

**TABLE 1:** Phage GenBank and SRA accession numbers and genome assembly results

Phage name	GenBank accession no.	SRA accession no.	Location (GPS coordinates)	Avg coverage (×)	No. of reads (thousands)	Cluster	Genome size (bp)	Genome ends	GC content (%)	No. of genes
Mercedes	MT498063	SRX9773838	34.938978N, 81.033121W	1,094	310.7	EA	40,230	Circular permuted	63.3	58
Leafus	MT498062	SRX9773837	34.940331N, 81.034923W	1,489	440.6	EA1	42,000	Circular permuted	63.4	63
Nebulous	MT451984	SRX9773839	34.9374N, 81.0308W	707	207.6	EA5	41,419	Circular permuted	64.3	57
Ixel	MT451983	SRX9773836	34.9407N, 81.0344W	1,263	361.2	EA11	40,556	Circular permuted	63.3	61

For all bioinformatics analyses and software, default parameters were used. The genome sequences were annotated to identify open reading frames and predicted protein functions using DNA Master v5.22.3 (8), Glimmer v3.02 (9), GeneMark v2.5 (10), Starterator (8), Phamerator v3 (11), hhPred v2.07 (12), and BLASTp v2.7.1 (13). All four phages have the typical genomic architecture seen in the *Microbacterium* phage cluster EA genomes (1). Genes in the 5′ half of the genome sequence are forward encoded and include sequences for a portal protein, scaffolding protein, a major capsid protein, two tail assembly chaperones (predicted to be expressed using a programmed translational frameshift in Mercedes, Nebulous, and Ixel), and a tape measure protein. The majority of the 3′ half of the genome sequences are encoded on the reverse strand and include coding for DNA Pol I, MazG-like protein, and thymidylate synthase. None of the genome sequences contained genes for tRNAs, integrases, or immunity repressors, and so these phages are predicted to solely use the lytic pathway for replication.

### Data availability.

The individual GenBank and SRA accession numbers are listed in Table 1. The actinobacteriophage sequencing BioProject accession number is PRJNA488469.

## References

[1] Jacobs-Sera D, Abad LA, Alvey RM, Anders KR, Aull HG, Bhalla SS, Blumer LS, Bollivar DW, Bonilla JA, Butela KA, Coomans RJ, Cresawn SG, D'Elia T, Diaz A, Divens AM, Edgington NP, Frederick GD, Gainey MD, Garlena RA, Grant KW, Gurney SMR, Hendrickson HL, Hughes LE, Kenna MA, Klyczek KK, Kotturi H, Mavrich TN, McKinney AL, Merkhofer EC, Moberg Parker J, Molloy SD, Monti DL, Pape-Zambito DA, Pollenz RS, Pope WH, Reyna NS, Rinehart CA, Russell DA, Shaffer CD, Sivanathan V, Stoner TH, Stukey J, Sunnen CN, Tolsma SS, Tsourkas PK, Wallen JR, Ware VC, Warner MH, Washington JM, Westover KM, Whitefleet-Smith JL, Wiersma-Koch HI, Williams DC, Zack KM, Hatfull GF. 2020. Genomic diversity of bacteriophages infecting Microbacterium spp. PLoS One 15:e0234636. doi:10.1371/journal.pone.0234636. 32555720 PMC7302621

[2] Jacobs-Sera D, Marinelli LJ, Bowman C, Broussard GW, Guerrero Bustamante C, Boyle MM, Petrova ZO, Dedrick RM, Pope WH, Modlin RL, Hendrix RW, Hatfull GF, Science Education Alliance Phage Hunters Advancing Genomics and Evolutionary Science Sea-Phages (SEA-PHAGES) program. 2012. On the nature of mycobacteriophage diversity and host preference. Virology 434:187–201. doi:10.1016/j.virol.2012.09.026. 23084079 PMC3518647

[3] Jordan TC, Burnett SH, Carson S, Caruso SM, Clase K, DeJong RJ, Dennehy JJ, Denver DR, Dunbar D, Elgin SCR, Findley AM, Gissendanner CR, Golebiewska UP, Guild N, Hartzog GA, Grillo WH, Hollowell GP, Hughes LE, Johnson A, King RA, Lewis LO, Li W, Rosenzweig F, Rubin MR, Saha MS, Sandoz J, Shaffer CD, Taylor B, Temple L, Vazquez E, Ware VC, Barker LP, Bradley KW, Jacobs-Sera D, Pope WH, Russell DA, Cresawn SG, Lopatto D, Bailey CP, Hatfull GF. 2014. A broadly implementable research course in phage discovery and genomics for first-year undergraduate students. mBio 5:e01051-13. doi:10.1128/mBio.01051-13. 24496795 PMC3950523

[4] Gordon D, Green P. 2013. Consed: a graphical editor for next-generation sequencing. Bioinformatics 29:2936–2937. doi:10.1093/bioinformatics/btt515. 23995391 PMC3810858

[5] Miller JR, Koren S, Sutton G. 2010. Assembly algorithms for next-generation sequencing data. Genomics 95:315–327. doi:10.1016/j.ygeno.2010.03.001. 20211242 PMC2874646

[6] Russell DA, Hatfull GF. 2017. PhagesDB: the actinobacteriophage database. Bioinformatics 33:784–786. doi:10.1093/bioinformatics/btw711. 28365761 PMC5860397

[7] Mavrich TN, Hatfull GF. 2017. Bacteriophage evolution differs by host, lifestyle and genome. Nat Microbiol 2:17112. doi:10.1038/nmicrobiol.2017.112. 28692019 PMC5540316

[8] Pope WH, Jacobs-Sera D. 2018. Annotation of bacteriophage genome sequences using DNA Master: an overview. Methods Mol Biol 1681:217–229. doi:10.1007/978-1-4939-7343-9_16. 29134598

[9] Delcher AL, Bratke KA, Powers EC, Salzberg SL. 2007. Identifying bacterial genes and endosymbiont DNA with Glimmer. Bioinformatics 23:673–679. doi:10.1093/bioinformatics/btm009. 17237039 PMC2387122

[10] Besemer J, Borodovsky M. 2005. GeneMark: Web software for gene finding in prokaryotes, eukaryotes and viruses. Nucleic Acids Res 33:W451–W454. doi:10.1093/nar/gki487. 15980510 PMC1160247

[11] Cresawn SG, Bogel M, Day N, Jacobs-Sera D, Hendrix RW, Hatfull GF. 2011. Phamerator: a bioinformatic tool for comparative bacteriophage genomics. BMC Bioinformatics 12:395. doi:10.1186/1471-2105-12-395. 21991981 PMC3233612

[12] Söding J, Biegert A, Lupas AN. 2005. The HHpred interactive server for protein homology detection and structure prediction. Nucleic Acids Res 33:W244–W248. doi:10.1093/nar/gki408. 15980461 PMC1160169

[13] Altschul SF, Gish W, Miller W, Myers EW, Lipman DJ. 1990. Basic local alignment search tool. J Mol Biol 215:403–410. doi:10.1016/S0022-2836(05)80360-2. 2231712

